# Clinical Experience with Dimethyl Fumarate and Natalizumab in Pregnant Women with Multiple Sclerosis: A Four-Patient Case Series

**DOI:** 10.1155/2024/7808140

**Published:** 2024-07-16

**Authors:** Satoshi Saito, Ryotaro Ikeguchi, Kazuo Kitagawa, Yuko Shimizu

**Affiliations:** Department of Neurology Tokyo Women's Medical University School of Medicine, 8-1, Kawadacho, Shinjukuku, Tokyo, Japan

## Abstract

Interferon *β* and glatiramer acetate are the disease-modifying drugs (DMDs) considered relatively safe for use in pregnant women with multiple sclerosis (MS); however, the safety profile of dimethyl fumarate (DMF) and natalizumab (NTZ) in this population remains inconclusive. Here, we present four cases of pregnant women with MS who were treated with DMF and NTZ (*n* = 2 patients, each) during their pregnancy and discuss our observations with the use of these drugs and the clinical courses of the patients. We retrospectively examined relapse of MS during pregnancy and after delivery; duration of exposure to DMDs; maternal, fetal, and neonatal adverse events; breastfeeding; and timing of resumption of DMDs. The two patients treated with DMF discontinued treatment 5 or 6 weeks after the discovery of pregnancy. DMF was resumed 1 week postpartum, and mixed breastfeeding was initiated. Brain magnetic resonance imaging in one patient 9 months after delivery revealed a new lesion; however, it was not classified as a clinical relapse. In two patients treated with NTZ, the dosing interval was extended to 6 weeks after the discovery of pregnancy. One patient discontinued NTZ at 30 weeks and the other at 25 weeks of gestation, as a slight restriction in fetal growth was observed owing to hyperemesis gravidarum. Both patients opted for formula feeding, and no relapse was observed within 1 year postpartum. Additionally, no abnormalities were observed in any of the patients during the perinatal period, and their development was normal. Investigation of drug safety in pregnant and parturient women primarily relies on registries, postmarketing surveillance, and case reports due to ethical limitations on conducting randomized controlled trials. Our findings demonstrated that DMF and NTZ were not contraindicated during pregnancy or the perinatal period in women with MS; nevertheless, vigilant monitoring is essential to ensure the safety of these drugs.

## 1. Introduction

Multiple sclerosis (MS) is an autoimmune disease affecting the central nervous system, commonly observed in women, particularly those of childbearing age [1]. A cohort study, excluding the use of disease-modifying drugs (DMDs), revealed that the average annual relapse rate (ARR) before pregnancy is 0.7 per woman, which declines to 0.2 in the third trimester of pregnancy and spikes to 1.2 at 3 months postpartum [2]. MS relapses in 31% of women during their pregnancy; however, the relapse rate gradually decreases in the subsequent period after childbirth [2]. Data from the MSBase registry on natalizumab (NTZ), fingolimod, and dimethyl fumarate (DMF) use and pregnancy-related relapses in an MS cohort after DMD dissemination showed a prepregnancy ARR of 0.29, decreasing to 0.19 in the third trimester, and then increasing to 0.59 (0.51–0.67) in the early postpartum period [3]. Continuation of NTZ treatment during pregnancy reduced relapse rates, whereas resuming DMDs with NTZ reduced postpartum relapse [3]. Moreover, relapse was less likely in breastfeeding women [3]; particularly, exclusive breastfeeding has been shown to reduce the risk of early postpartum relapse [4]. Hughes et al. demonstrated that administration of DMDs 2 years before pregnancy reduced the risk of relapse at 3 months postpartum by 45%, highlighting the importance of suppressing disease activity before delivery [5]. Glatiramer acetate and interferon *β* (IFN*β*) are the DMDs considered relatively safe for use during pregnancy and breastfeeding [6] and have moderate efficacy for preventing relapse. However, DMF and NTZ exhibit stronger relapse-inhibitory effects than glatiramer acetate and IFN*β*, with NTZ being the strongest [7]. Japanese product documents on DMF suggest that the transfer of DMF to human milk is unclear [8], and the continuation or discontinuation of breastfeeding with DMF should be decided by taking the therapeutic benefits of breastfeeding into account. However, regarding breastfeeding, the Japanese product document for NTZ does not provide data on the time required for NTZ to disappear from breast milk. However, considering the elimination half-life of NTZ in plasma, breastfeeding should be discontinued during natalizumab treatment and for 12 weeks after the final dose [9]. This is inconsistent with the recommendations of the Food and Drug Administration (FDA) [10] and the Drugs and Lactation Database (LactMed) [11], potentially necessitating revision.

Several clinical studies have investigated the effects of DMDs on pregnancy and breastfeeding; however, the number of detailed case reports on the use of DMDs during the perinatal period in Japan is scarce. Consequently, the utilization of these drugs in Japan relies partly on established guidelines and partly on the discretion of attending physicians. In this study, we discuss four cases of pregnant women who were treated with DMF and NTZ during pregnancy and breastfeeding. We describe the safety profile and our observations with the use of these drugs with respect to the incidence of relapse in mothers during pregnancy and after delivery, duration of exposure to DMDs during pregnancy, mode of delivery, incidence of congenital anomalies in neonates, timing of postpartum initiation of DMDs, and breastfeeding.

## 2. Case Presentation

This report included four pregnant women treated with DMF (*n* = 2) or NTZ (*n* = 2) and followed up for at least 1 year postpartum on an outpatient basis at our hospital between 2015 and 2023. We retrospectively examined the incidence of relapse associated with pregnancy or delivery; duration of exposure to DMDs; maternal, fetal, and neonatal adverse events; breastfeeding; and timing of reinstitution of DMD therapy. The clinical characteristics of the four women are shown in Table 1.

### 2.1. Case 1

**Figure 1:**
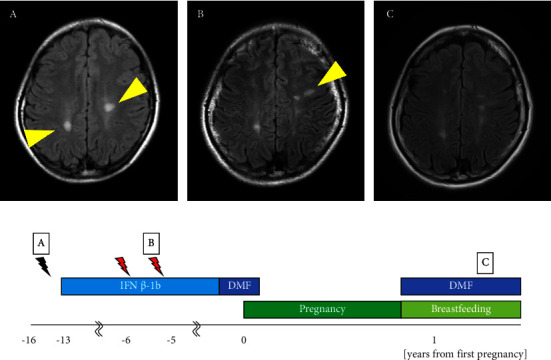
Clinical course of Patient 1. Relationship between duration of disease-modifying drug therapy (IFN*β* ⟶ DMF) and relapse during pregnancy and breastfeeding. MRI findings, represented by yellow triangles, illustrate multiple sclerosis at 15 years (A), 24 years (B), and 9 months after delivery (C). Black lightning indicates an asymptomatic new lesion on MRI. The red lightning indicates clinical relapse. IFN, interferon; DMF, dimethyl fumarate, 480 mg/d.

The patient was a 31-year-old woman. At the age of 14 years, she noticed muscle weakness in her right lower limb and sensory disturbances in the right hand. Brain magnetic resonance imaging (MRI) revealed oval plaques in the white matter around the lateral ventricle with ring enhancement on gadolinium-enhanced imaging, leading to the suspicion of MS. At the age of 15 years, follow-up brain MRI revealed increased asymptomatic plaques with a black hole in the white matter of the right parietal lobe and the white matter around the left lateral ventricle on T1-weighted imaging (Figure 1(A)). At the age of 17 years, the cerebrospinal fluid tested positive for oligoclonal bands and showed an elevation in the immunoglobulin G (IgG) index to 0.98. She met the McDonald diagnostic criteria [12] and was diagnosed with MS, after which treatment with IFN*β*-1b was initiated. MS relapse occurred in the left optic nerve at 23 years of age, in the right optic nerve at 24 years of age, and in a subcortical lesion in the left frontal lobe at 25 years of age (Figure 1(B)). IFN*β*-1b was continued, and subsequent brain MRI did not show any evidence of new plaque formation. At the age of 30 years, IFN*β*-1b was switched to 480 mg/d of DMF. She became pregnant in the same year with an expanded disability status scale (EDSS) score of 1.0 and discontinued DMF treatment at 5 weeks of gestation. DMF was administered for 4 months after its initiation. She delivered vaginally at 39 weeks of gestation and resumed DMF while subjecting her child to mixed breastfeeding, with an EDSS score of 1.0. At 2 and 6 months postpartum, MRI showed no new lesions suggestive of relapse compared with 1 month before pregnancy. The child developed normally and had no serious infections (Figure 1(C)).

### 2.2. Patient 2

**Figure 2:**
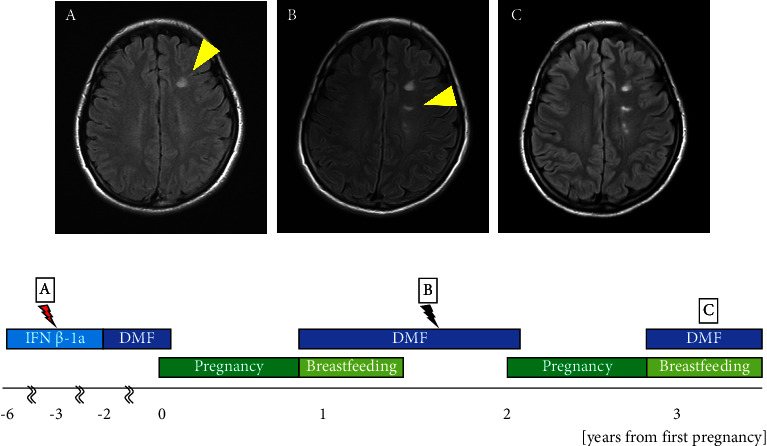
Clinical course of Patient 2. Relationship between duration of disease-modifying drug therapy (IFN*β* ⟶ DMF) and relapse during pregnancy and breastfeeding. MRI findings, represented by yellow triangles, illustrate multiple sclerosis at 28 years (A), 9 months after the first delivery (B), and 9 months after the second delivery (C). Black lightning indicates an asymptomatic new lesion on MRI. The red lightning indicates clinical relapse. IFN: interferon, DMF: dimethyl fumarate 240 mg/d.

The patient was a 34-year-old woman. At the age of 25, she noticed pain in the left eye accompanied by a left visual field defect. Brain MRI revealed gadolinium enhancement in the left optic nerve, multiple plaques in the cortex of the right parietal lobe, and white matter in the left frontal and temporal lobes. Cerebrospinal fluid testing showed an elevation in the IgG index to 0.81 and positivity for oligoclonal bands, which met the McDonald diagnostic criteria [12]. The patient was diagnosed with MS, and treatment with IFN*β*-1a was initiated. She was referred to our department at the age of 26. At the age of 28 years, the MS relapsed in the right optic nerve. Brain MRI revealed a new demyelinating lesion in the white matter of the left frontal lobe (Figure 2(A)). At the age of 29 years, IFN*β*-1a was replaced with DMF at 480 mg/d. The dose was reduced to 240 mg/d owing to a decline in the lymphocyte count. At the age of 31 years, DMF was discontinued at 6 weeks of gestation. The patient's EDSS score at conception was 0.0. At the age of 32 years, she was temporarily hospitalized for threatened premature delivery at 29 weeks of gestation but delivered vaginally at 40 weeks. DMF was resumed immediately after delivery. Breastfeeding was withheld for 4 h after the oral administration of DMF, and mixed breastfeeding was initiated. A follow-up brain MRI performed 9 months postpartum showed a new asymptomatic demyelinating lesion in the left lateral ventricle compared to 4 months before pregnancy (Figure 2(B)). Mixed breastfeeding was continued for 1 year and 3 months postpartum. The child developed normally and had no serious infections. At the age of 33 years, she became pregnant with her second child and discontinued DMF at 6 weeks of gestation, with an EDSS score of 0.0. At the age of 34 years, she delivered vaginally at 39 weeks of gestation. DMF was resumed 7 d postpartum, and mixed breastfeeding was initiated with an EDSS score of 0.0. No relapse was observed at 2 or 9 months postpartum (Figure 2(C)). The second child developed normally and had no serious infections.

### 2.3. Patient 3

**Figure 3:**
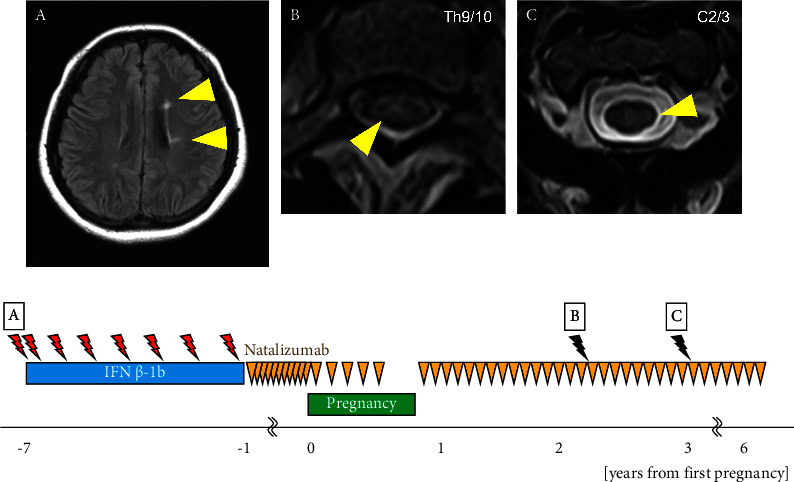
Clinical course of Patient 3. Relationship between duration of disease-modifying drug therapy (IFN*β* ⟶ NTZ) and relapse during pregnancy and breastfeeding. MRI findings, represented by yellow triangles, illustrate multiple sclerosis at 25 (A), 34 (B), and 35 years (C). Orange triangles indicate an intravenous infusion of 300 mg natalizumab. Black lightning indicates an asymptomatic new lesion on MRI. The red lightning indicates clinical relapse. IFN: interferon, NTZ: natalizmub.

The patient was a 38-year-old woman. At the age of 25, she noticed numbness in the left hand and diplopia. A cervical spine MRI revealed a lesion suggestive of demyelination at the C2/3 level. Brain MRI revealed multiple ovoid lesions in the white matter around the lateral ventricle (Figure 3(A)). The patient met the McDonald diagnostic criteria [12], leading to a diagnosis of MS, and treatment with IFN*β*-1b was initiated. In the same year, she experienced sensory disturbance on the left side of the neck, and cervical spine MRI revealed a new lesion with gadolinium enhancement at the C1/2 level. At the age of 26 years, she noticed a sensory disturbance in the chest, and thoracic spine MRI showed a demyelinating lesion with gadolinium enhancement at Th6/7. At the age of 27 years, she developed gaze-evoked nystagmus of the right eye and gait disturbance. Brain MRI revealed demyelinating lesions with gadolinium enhancement in the white matter of the right frontal lobe and left middle cerebellar peduncle. Clinical relapses occurred once at 28 years, twice at 29 years, and once at 30 years. At the age of 31 years, IFN*β*-1b was switched to NTZ 300 mg intravenously administered every 4 weeks. She conceived at the age of 32 years with an EDSS score of 1.0, and the NTZ dosing interval was extended to 6 weeks. NTZ was discontinued at 25 weeks of gestation because hyperemesis gravidarum led to a slight restriction of fetal growth. The patient delivered vaginally at 39 weeks of gestation. NTZ was resumed 5 d postpartum with an EDSS score of 1.0, and feeding with artificial milk was initiated. At 17 months postpartum, thoracic spine MRI showed new asymptomatic lesions at the Th5/6 and Th9/10 thoracic spine levels compared with those at 4 months postpartum (Figure 3(B)). At the age of 35 years, cervical spine MRI revealed a new asymptomatic lesion at C2/3 (Figure 3(C)). The child developed normally and had no serious infections.

### 2.4. Patient 4

**Figure 4:**
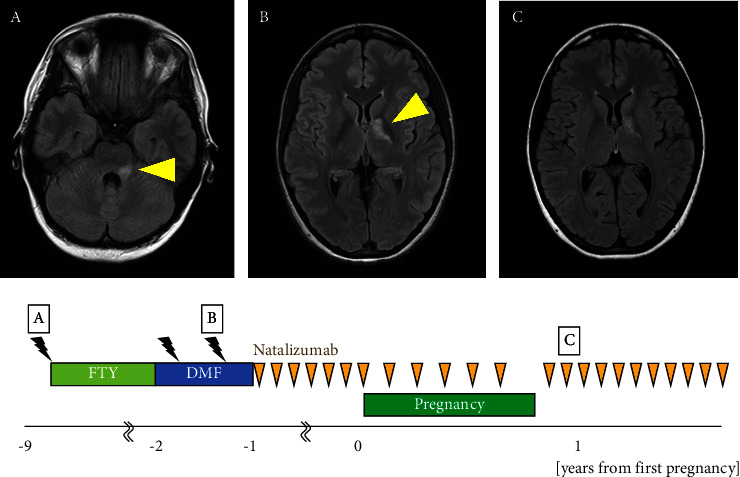
Clinical course of Patient 4. Relationship between duration of disease-modifying drug therapy (FTY ⟶ DMF ⟶ NTZ) and relapse during pregnancy and breastfeeding. MRI findings, represented by yellow triangles, illustrate multiple sclerosis at 26 years (A), 33 years (B), and 1 year and 2 months after delivery (C). Orange triangles indicate an intravenous infusion of 300 mg natalizumab. Black lightning indicates an asymptomatic new lesion on MRI. DMF, dimethyl fumarate; 480 mg/d; FTY, fingolimod 0.5 mg/d; NTZ, natalizmub.

The patient was a 36-year-old woman. At the age of 26 years, she developed dysarthria, left facial paralysis, nystagmus, and gait disturbance. Brain MRI revealed demyelinating lesions in the left pons and middle cerebellar peduncle (Figure 4(A)). Cerebrospinal fluid testing showed an IgG index score of 0.41 and negativity for oligoclonal bands. At the age of 27 years, brain MRI showed enlarged demyelinating lesions in the left pons and middle cerebellar peduncle and a new demyelinating lesion around the left lateral ventricle, which met the McDonald diagnostic criteria [12], and the patient was diagnosed with MS. At the age of 27 years, treatment with fingolimod 0.5 mg/d was initiated, and no relapse occurred until the age of 33 years. At 33 years of age, fingolimod was replaced with DMF at 480 mg/d because the patient expressed her intent to become pregnant. Four months after the switch, a follow-up brain MRI showed a demyelinating lesion in the left globus pallidus (Figure 4(B)). Four months later, the lesion on the left globus pallidus had enlarged. At the age of 33 years, she was referred to our department with an EDSS score of 1.0. At the age of 34 years, the medication was switched to NTZ 300 mg intravenously every 4 weeks. At 35 years of age, she became pregnant, and the NTZ dosing interval was extended to 6 weeks. NTZ was discontinued at 30 weeks, and the patient delivered at 39 weeks of gestation. NTZ was resumed 14 d postpartum with an EDSS score of 1.0, and feeding with artificial milk was initiated. No new lesions were observed at 1 year or 2 months postpartum (Figure 4(C)). The child developed normally and had no serious infections.

## 3. Discussion

MS occurs predominantly in women, and the most common age of onset coincides with the childbearing age. Before the advent of immunoregulatory therapy, it was common to avoid pregnancy because of the risk of relapse associated with pregnancy and delivery. However, with the widespread use of DMDs and monoclonal antibody drugs, relapses associated with pregnancy complicated by MS have become preventable, allowing more women to pursue pregnancy. However, the increased options for DMDs have complicated the treatment and management of pregnant women with MS. In this study, we report four cases of pregnant women with MS who achieved favorable outcomes with DMF or NTZ. We discussed the detailed clinical courses and effects of these two drugs on mothers and children.

DMF suppresses inflammatory reactions in the peripheral immune and central nervous systems by activating nuclear factor (erythroid-derived 2)-like factor 2-dependent transcription. It exerts a disease-modifying effect on MS via antioxidant activity that protects neurons from damage [13]. Studies have reported that the ARR decreased significantly by 49% in patients treated with DMF 2 years posttreatment compared to that in the placebo group [14]. Furthermore, it has also been shown that the intrapartum ARR decreases with preconception use of DMF, while it spikes postdelivery in all groups treated with DMDs [3]. Animal experiments using DMF showed that neither fetal teratogenicity nor disorder occurred at doses 11 and 16 times higher than the clinical dose; however, its administration during gestation and lactation had adverse effects on the survival, growth, maturation, and neuroethological function of the offspring [8]. Observational postmarketing studies have reported a spontaneous abortion incidence similar to that of the general population, but caution is warranted in interpreting this finding due to the lack of well-controlled clinical studies [8]. Both Japanese regulatory authorities and the European Medicines Agency (EMA) recommend administering DMF to pregnant or potentially pregnant women only if the therapeutic benefits outweigh the risks [8, 15]. The United States FDA indicates that DMF may be detrimental to the fetus despite the lack of sufficient data [16]. In this study, in patients 1 and 2, pregnancy was detected approximately 6 weeks after DMF initiation. Concordant with this, an international registry (TecGistry; NCT01911767) evaluating DMF-exposed pregnancy and infant outcomes in 345 pregnant women with MS reported that fetal exposure to DMF up to the first trimester of pregnancy had no adverse effects on the birth rate, fetal growth, fetal mortality, or the incidence of congenital anomalies [17]. In the same registry, 379 pregnant women exposed to DMF for a median duration of 5 weeks of gestation showed no adverse pregnancy outcomes, and the rates of congenital disabilities, preterm births, and spontaneous abortions were consistent with those in the general population [18]. In our patient, DMF was discontinued in the first trimester with no adverse events.

The EMA recommends that oral administration of DMF should be discontinued during breastfeeding because it is unknown whether DMF and its metabolites are secreted into breast milk [15], while the FDA has proposed a comprehensive risk-benefit assessment for the continuation of DMF during breastfeeding [16]. According to the Japanese package inserts, DMF should be administered if its benefits outweigh its risks [8]. In an overseas case series investigating the transfer of DMF into breast milk during treatment, the relative infant doses were 0.019% and 0.007% in two patients, far below the critical level of 10% [19]. A study involving 22 patients who resumed DMF 1 month postpartum observed no adverse events in their infants [20]. According to LactMed, DMF is not detected in the plasma because it is rapidly converted to monomethyl fumarate, which is an active metabolite with a half-life of approximately 1 h, suggesting low levels of DMF and its metabolite in breast milk and minimal risk to breastfed infants [21]. According to the Japanese package insert of DMF, its half-life is 0.86 ± 0.85 h after an oral dose of 120 mg twice daily, whereas it is 0.66 ± 0.22 h after an oral dose of 240 mg twice daily, indicating a rapid decline in maternal blood concentration after oral administration [8]. Based on the above-mentioned evidence and the benefits of DMF, we continued mixed breastfeeding after obtaining consent from our patients. However, current expert opinion advises against breastfeeding while on DMF until more safety data is available [22]. Therefore, further evidence from patient cohorts is necessary to confirm the safety of this drug during breastfeeding.

NTZ is a monoclonal antihuman IgG4 antibody to *α*4 integrin, an adhesion molecule. NTZ exerts a disease-modifying effect on MS by inhibiting lymphocyte migration to extravascular tissues [23]. Transfer of IgG across the placenta increases starting at 16 weeks of gestation, peaking at 26 weeks [24, 25]. *α*4 integrins are also involved in the migration of erythroid progenitors and pre-B cells beneath the stroma [26]. Therefore, NTZ can cause hematological abnormalities with frequencies ranging from 6% to 77% [27, 28]. However, continuing NTZ during pregnancy reduces the odds of relapse by 0.76 per month [3], while its discontinuation before pregnancy or during the first trimester has been shown to cause significant clinical disability in 10% of women at 1 year postpartum [29]. Adapting dosing by extending intervals to 6 weeks during pregnancy and discontinuing at 30 weeks effectively mitigates these risks [30]. In Patients 3 and 4, NTZ was administered at 4 weeks before pregnancy, and the dosing interval was extended to 6 weeks during pregnancy. Patient 4 continued NTZ until 30 weeks of gestation, whereas Patient 3 discontinued it at 25 weeks of gestation because hyperemesis gravidarum led to a slight restriction in fetal growth. Both the patients delivered vaginally at 39 weeks of gestation without complications. Although it resumed soon after delivery, no adverse events were observed in either the mothers or their children. In Patient 3, a follow-up cervical spine MRI performed 18 months postpartum revealed a new, asymptomatic demyelinating lesion. However, as NTZ is reportedly associated with a risk of rebound after discontinuation, the decision to discontinue the drug should be made after careful deliberation [31]. The “Association of British Neurologists guidelines” recommend that NTZ should not be suspended before pregnancy or the first trimester of pregnancy but should be continued at an extended dosing interval of 8 weeks until approximately 34 weeks of gestation in cases with high disease activity and serious anticipated relapse [32]. In a report of 106 patients who continued NTZ treatment after 30 weeks of gestation, early postpartum resumption decreased the postpartum relapse rate but increased the risk of neonatal hematological abnormalities [33]. Another study also reported that reinstitution of NTZ within 8 d of delivery in women at high risk of postpartum relapse reduced the ARR after delivery in five of six women compared to the relapse rate before the initiation of antepartum NTZ therapy [34]. Therefore, we continued treatment with NTZ until 30 weeks of gestation after accounting for the risks of transient hematological abnormalities in neonates due to drug exposure during pregnancy and its benefits to the mother.

According to the Japanese labeling, because NTZ is reportedly transferred to human breast milk, breastfeeding should be discontinued during treatment and for 12 weeks after the final dose [9]. The EMA indicates that breastfeeding should be avoided [35], whereas the FDA permits it depending on a risk-benefit assessment [10]. According to LactMed, NTZ, a large protein with a molecular weight of approximately 149,000 Da, is likely to be partially destroyed in the gastrointestinal tract of infants, and its absorption by infants is minimal [11]. A study of three patients in whom NTZ was continued during breastfeeding demonstrated that NTZ was detected in the breast milk; however, the relative infant dose was 0.5%, which was far below the critical level of 10%. Moreover, NTZ was not detected in the serum of infants [36]. Furthermore, expert guidelines do not recommend avoiding breastfeeding during treatment with NTZ [11, 29, 36], whereas a recent review indicated that breastfeeding could be allowed after NTZ [22].

## 4. Conclusion

In this study, we discuss the safety of DMF and NTZ during pregnancy and breastfeeding complicated with MS. Management protocols in women with MS during pregnancy and breastfeeding differ slightly between Japan, the United States, and Europe, and the level of evidence for each interpretation is low. Therefore, consideration of the administration of DMDs that are not contraindicated in pregnant women is essential to maintain remission in cases of pregnancy, especially in patients with high disease activity associated with a high risk of relapse. In our cases, exposure to DMF and NTZ during pregnancy did not cause any adverse events in either the mother or fetus. No serious relapse was observed during early pregnancy, and the development of the children was normal. However, the long-term effects of these DMDs in pregnant women with MS are unknown and have not been fully elucidated. Therefore, during family planning, shared decision-making should be implemented in consideration of the patient's wishes after sufficient evaluation of the disease activity in each patient, including the selection of DMDs, timing of discontinuation of DMDs in preparation for pregnancy, duration of treatment, timing of postpartum resumption of DMDs, and method of breastfeeding.

## Figures and Tables

**Table 1:** Summary of the characteristics of patients with multiple sclerosis during pregnancy.

	Patient 1	Patient 2^*∗*^	Patient 3	Patient 4
Age at onset (year)	17	25	25	27
Age at delivery (year)	30	31.33	32	35
Preconception EDSS score	1.0	0.0	1.0	1.0
Postpartum EDSS score	1.0	0.0	1.0	1.0
Relapse before the first year of pregnancy	0	0.0	0	0
Type of DMDs	IFN*β* ⟶ DMF	IFN*β* ⟶ DMF	IFN*β* ⟶ NTZ	FTY ⟶ DMF ⟶ NTZ
Discontinuation of DMDs after conception (weeks)	5	6.6	25	30
Delivery (weeks)	39	40.39	39	39
Delivery complication	None	None	None	None
Breastfeeding	Mixed	Mixed, mixed	None	None
Resuming DMDs after delivery (weeks)	1	1.1	1	2
Newborn weight (g)	2942	3455.3413	2582	3014
Congenital anomaly	None	None, none	None	None
Hemorrhagic abnormality	None	None, none	None	None
Infant serious infection	None	None	None	None
New MRI lesion after delivery within 1 year	0	1.0	0	0
Clinical relapse after delivery within 1 year	0	0, 0	0	0

EDSS, expanded disability status scale; DMDs, disease-modifying drugs; IFN*β*, interferon-*β*; DMF, dimethyl fumarate; NTZ, natalizumab; FTY, fingolimod. ^*∗*^Patient 2 carried two babies.

## Data Availability

The data are available from the first author upon reasonable request.
